# The influence of *APOE^ε4^* on the pTau interactome in sporadic Alzheimer’s disease

**DOI:** 10.1002/alz.091601

**Published:** 2025-01-03

**Authors:** Manon Thierry, Jackeline Ponce, Mitchell Marta Ariza, Manor Askenazi, Arline Faustin, Dominique Leitner, Geoffrey Pires, Evgeny Kanshin, Eleanor Drummond, Beatrix Ueberheide, Thomas Wisniewski

**Affiliations:** ^1^ NYU Grossman School of Medicine, New York, NY USA; ^2^ Biomedical Hosting LLC, Arlington, MA USA; ^3^ The University of Sydney, Sydney, NSW Australia

## Abstract

**Background:**

*APOE^ε4^* is the major genetic risk factor for sporadic Alzheimer’s disease (AD). Although *APOE^ε4^* is well known to promote Aβ pathology, recent data also support an effect of *APOE* polymorphism on phosphorylated Tau (pTau) pathology.

**Method:**

To elucidate this potential effect, the pTau interactome was analyzed across *APOE* genotypes in the frontal cortex of 10 advanced AD cases (n = 5 *APOE* ^ε3/ε3^ and n = 5 *APOE^ε4/ε4^*), using a combination of anti‐pTau PHF1 (pS396/pS404) immunoprecipitation and mass spectrometry. This proteomic approach was complemented by a neuropathological analysis of anti‐pTau PHF1 and anti‐Aβ 4G8 immunohistochemistry, performed in the frontal cortex of 21 advanced AD cases (n = 11 *APOE* ^ε3/ε3^ and n = 10 *APOE^ε4/ε4^*).

**Result:**

Our dataset includes 1130 and 1330 proteins enriched in IP_PHF1_ samples from *APOE* ^ε3/ε3^ and *APOE^ε4/ε4^* groups (FC ≥ 1.50, IP_PHF1_ *versus* IP_IgG ctrl_). We identified 80 and 68 proteins as pTau interactors in *APOE* ^ε3/ε3^ and *APOE^ε4/ε4^* groups, respectively (SAINT score ≥ 0.80; FDR ≤ 5%). A total of 47/80 proteins were identified as pTau interactors only for *APOE^ε3/ε3^* cases. Functional enrichment analyses showed that they were significantly associated with the nucleoplasm compartment and involved in RNA processing. In contrast, 35/68 proteins were identified as pTau interactors only for *APOE^ε4/ε4^* cases. They were significantly associated with the synaptic compartment and involved in cellular transport. A comprehensive characterization of Tau pathology in the frontal cortex showed a higher density of plaque‐associated neuritic crowns, made of dystrophic axons and synapses, in *APOE^ε4^* carriers. Cerebral amyloid angiopathy was more frequent and severe in *APOE^ε4/ε4^* cases.

**Conclusion:**

Our study supports an influence of *APOE* genotype on pTau subcellular location in the human brain. These results are consistent with a facilitation of pTau progression in Aβ‐affected brain regions of *APOE^ε4^* carriers, paving the way to the identification of potential new therapeutic targets.

